# *Rattus norvegicus* (Rodentia: Muridae) Infected by *Leishmania* (*Leishmania*) *infantum* (syn. *Le. chagasi*) in Brazil

**DOI:** 10.1155/2014/592986

**Published:** 2014-02-23

**Authors:** Fabiana de Oliveira Lara-Silva, Ricardo Andrade Barata, Érika Monteiro Michalsky, Eduardo de Castro Ferreira, Maria Olímpia Garcia Lopes, Aimara da Costa Pinheiro, Consuelo Latorre Fortes-Dias, Edelberto Santos Dias

**Affiliations:** ^1^Laboratório de Leishmanioses, Centro de Pesquisas René Rachou, Fundação Oswaldo Cruz, Belo Horizonte, MG, Brazil; ^2^Laboratório de Parasitologia, Departamento de Ciências Biológicas, Universidade Federal dos Vales do Jequitinhonha e Mucuri, Diamantina, MG, Brazil; ^3^Fundação Oswaldo Cruz, MS, Brazil; ^4^Biodiversity Salvation, Belo Horizonte, MG, Brazil; ^5^Secretaria Municipal de Saúde, Governador Valadares, MG, Brazil; ^6^Laboratório de Enzimologia Aplicada, Diretoria de Pesquisa e Desenvolvimento, Fundação Ezequiel Dias, Belo Horizonte, MG, Brazil

## Abstract

In the present study we surveyed the fauna of phlebotomine sand flies and small mammals in peridomestic areas from a Brazilian municipality where the American cutaneous leishmaniasis (ACL) is endemic. A total of 608 female phlebotomine sand flies were captured during nine months in 2009 and 2010. Seven different species were represented with 60% of them being *Lutzomyia intermedia* and *Lu. whitmani*, both incriminated vectors of ACL. *Lu. longipalpis*, a proven vector of visceral leishmaniasis (VL) was also captured at high proportion (12.8%). Genomic DNA analysis of 136 species-specific pools of female sand flies followed by molecular genotyping showed the presence of *Leishmania infantum* DNA in two pools of *Lu. longipalpis*. The same *Leishmania* species was found in one blood sample from *Rattus norvegicus* among 119 blood and tissue samples analysed. This is the first report of *Le. infantum* in *R. norvegicus* in the Americas and suggests a possible role for this rodent species in the zoonotic cycle of VL. Our study coincided with the reemergence of VL in Governador Valadares.

## 1. Introduction

Leishmaniases are a complex of parasitic diseases caused by flagellated protozoa belonging to the genus *Leishmania* Ross, 1903. In Brazil, they are primarily transmitted to humans through the bites of *Lutzomyia* (Diptera: Psychodidae) phlebotomine sand flies. Domestic and synanthropic hosts play a role in the peridomestic transmission of those diseases due to host and insect vector adaptation to new habitats created by human action [1].

In the 80's, human cases of the American cutaneous leishmaniasis (ACL) were reported in 19 of the 26 states of Brazil. In 2003, autochthonous cases were confirmed in all of them [2, 3]. Among the seven *Leishmania* species known to be causative agents of ACL in our country, *Leishmania* (*Viannia*) *braziliensis*, *Le*. (*V.*) *guyanensis*, and* Le*. (*Leishmania*) *amazonensis* are the most important ones [4]. Reports of wild reservoirs infected by *Leishmania* spp. emphasize the zoonotic character of ACL [5] and increased the importance of studies of domestic and peridomestic animals as spreading agents of the parasite to humans. A number of rodent, marsupial, and wild canid species were identified as natural hosts and potential reservoirs for *Leishmania* spp. [6, 7].

Our study aimed at a better understanding of ACL in an urban area where it is endemic, with identification of the phlebotomine sand fly vector, hosts, and circulating *Leishmania* species. The area was chosen based on the high numbers (241 cases between 2001 and 2006) of human cases of ACL in the last decade [8] and on the peculiar pattern of urban transmission in that region, since most human cases of ACL are registered in residents from the urban area with little or no contact with forested environments [9]. A set of features in the districts selected in our study are known to favor the occurrence of sand flies and mammalian reservoirs of *Leishmania*: domestic animal shelters very close to human dwellings, lack of basic hygienic conditions around the houses, and close location to forest remnants [10].

## 2. Material and Methods

### 2.1. Study Area and Phlebotomine Sand Fly Captures

**Figure 1:**
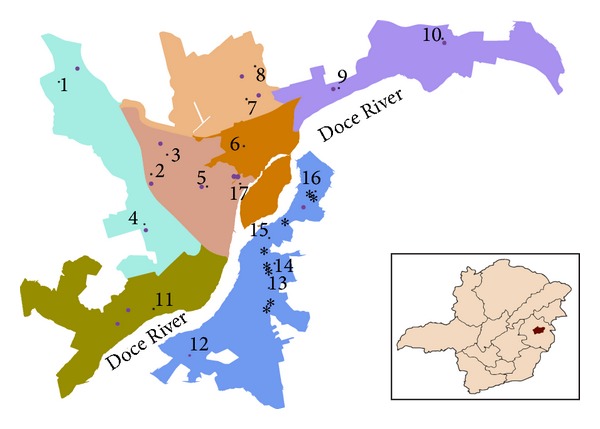
Geographical location of Governador Valadares municipality in the state of Minas Gerais, Brazil. Sites of capture of phlebotomine sand flies (pink circles) and small mammals trapping (black stars) in the districts under study (identified with black dots and numbers). *Lu. longipalpis* samples infected by *Le. infantum* were captured at sites 3 and 15; *R. norvegicus* equally infected was captured at site 14.

The municipality of Governador Valadares (18°51′12′′S, 41°56′42′′W) is located in the administrative region of the *Vale do Rio Doce* (Doce River Valley) in the Brazilian state of Minas Gerais (Figure 1). The city is an important socioeconomic pole of the region with 2349 Km^2^ of area and 260,000 inhabitants.

Nonsystematic entomological captures were performed in 17 districts of Governador Valadares (Figure 1) based on previous records of human cases of ACL. HP traps [11] were installed in the peridomiciles from 4:00 p.m. to 8:00 a.m., for two consecutive days during nine months (March/April/May/October/November/December of 2009; January/February/March of 2010). The captured female phlebotomine sand flies were stored in 6% DMSO under liquid nitrogen. After dissection for species identification, a varying number of individuals from the same species and capture site ware pooled and tested for the presence of *Leishmania *spp. DNA.

### 2.2. Small Mammal Captures (Reservoir)

Six captures were performed bimonthly between January and December 2008, using forty Tomahawk traps baited with banana and cod liver oil. The traps were distributed in eight sites in the peridomiciles (two traps per site) from 4:00 p.m. to 8:00 a.m. for three consecutive nights. The captures were repeated in January and March 2010. Blood, ear and tail skins, liver and spleen samples, and bone marrow aspirates were collected from the captured animals. The blood samples were collected in EDTA. Other tissue samples were immediately used to prepare slide imprints, which were stained with Giemsa after methanol fixation or stored in microtubes with saline containing antibiotics (100 *μ*g/mL of streptomycin and 500 U/mL of penicillin). The field captures were previously licensed by the Brazilian Institute of Environment and Renewable Natural Resources (IBAMA, license 154/07).

### 2.3. Extraction of Genomic DNA

Total genomic DNA was extracted from the female sand flies and from mammalian tissues using the Illustra Tissue and Cells genomicPrep Mini Spin kit (GE HealthCare) according to the manufacturer's instructions.

### 2.4. Amplification of Constitutive Genes

Genus-specific primers for *Lutzomyia* (5Llcac 5′ GTG GCC GAA CAT AAT GTT AG 3′ e 3Llcac 5′ CCA CGA ACA AGT TCA ACA TC 3′) were used to amplify the IVS6 region (cacophony) of the phlebotomine sand fly DNA [12]. For small mammals, the DNA amplification was performed with primers (5′ TCC AAC ATC ACC ACC ACT GAG TGG AC 3′ and 5 AAG AAA TCG AGG GTG GAC TGG CC 3′) for the IRBP gene [13]. All the amplifications were performed with the PureTaq Illustra Ready-To-Go PCR Beads (GE Healthcare) for 35 cycles under annealing temperature of 55°C for 30 sec, in a Perkin-Elmer thermocycler GeneAmpPCRSystem-2400. Negative (no DNA) and positive (*Lutzomyia* or dog skin DNA) controls were included in every set of reactions. The amplified samples were submitted to electrophoresis on 2% agarose gels containing ethidium bromide.

### 2.5. *Leishmania* Nested PCR (LnPCR) Targeted to the SSUrRNA Gene

A first amplification step was performed with specific (R221 and R332) primers for the order Kinetoplastida but not exclusively for *Leishmania* [14, 15]. The amplifications conditions were denaturation at 94°C for 5 min, followed by 30 cycles of 30 seg at 94°C, 60°C, and 72°C, and a final extension at 72°C for 5 min. The resulting amplification product of 603 bp was used as template in a second reaction in the presence of *Leishmania*-specific (R223 and R333) primers [14, 15]. The reaction conditions were the same as before except for an increased annealing temperature of 65°C. The final reaction resulted in a 353 bp fragment that was visualized after electrophoresis on 2% agarose gel and ethidium bromide staining. For the amplifications we used the pureTaq Illustra Ready-To-Go PCR Beads kit (GE Healthcare) and every set of reactions included a negative (no DNA) and a positive (*Leishmania braziliensis* MHOM/BR/75/M2903DNA) control.

### 2.6. DNA Sequencing for *Leishmania* Species Identification

LnPCR amplified fragments were extracted from agarose gels by the QIAquick PCR Purification kit (QIAGEN). Sequencing reactions were prepared with 4 *μ*L of PREMIX (BigDye Terminator v3.1 kit), 3.2 pmol of primer in 1 *μ*L and, 5 *μ*L of the LnPCR product. PCR amplification was performed with 30 cycles of 95°C for 20 sec, 55°C for 15 sec, and 60°C for 1 min. For each sample, the sequencing reactions were prepared with forward and reverse primers in a MegaBACE 1000 DNA System. Bioinformatics analysis of the sequences was performed using the Lasergene sequence analysis (DNASTAR) and the BIOEDIT softwares.

### 2.7. Minimum Rate of Infection

The minimum rate of natural infection (TMI) was calculated by dividing the number of positive pools of a given species by the total number of individuals of the same species ×100.

## 3. Results

During the study period, we captured 608 female phlebotomine sand flies from seven different species (Table 1) with almost 60% of them being *Lu. intermedia* and *Lu. whitmani*, both incriminated vectors in the transmission of ACL. *Lu. longipalpis*, proven vector of visceral leishmaniasis (VL), was also captured at high proportion (12.8%). The number of species-specific pool samples prepared for molecular analysis is listed in Table 1.

**Figure 2:**
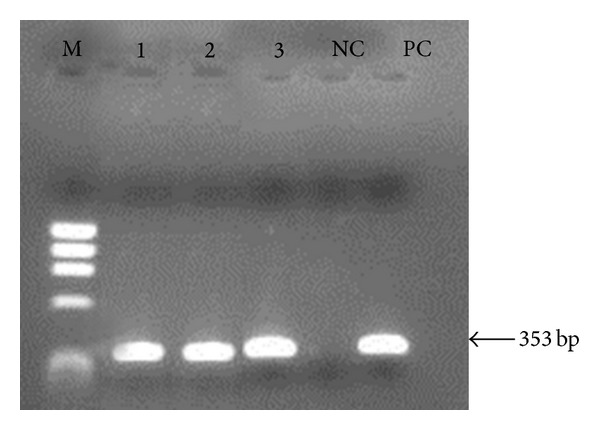
Agarose gel electrophoresis of the amplification products from LnPCR for the SSUrRNA gene. Samples: M. ΦX174/*Hae*III DNA digest; (1) *Lu. longipalpis* genomic DNA (GV05); (2) *Lu. longipalpis* genomic DNA (GV09); (3) *R. norvegicus* blood DNA (GV017); NC. Negative control (no DNA); PC. Positive control (*Le. braziliensis *MHOM/BR/74/M2930) DNA.

The extraction process of phlebotomine sand fly DNA was validated through amplification of a cacophony gene. Every pool sample of sand fly DNA showed the expected 220 bp band due to amplification of the IVS6 region (data not shown). After LnPCR, we observed the characteristic 353 bp fragment of *Leishmania* genus in two of the 136 pool samples of phlebotomine sand fly DNA analyzed (Figure 2). Both of them corresponded to *Lu. longipalpis* pool samples and their capture sites are identified in Figure 1. Thus, the TMI of natural *Leishmania* spp. infection in *Lu. longipalpis* in Governador Valadares was calculated as 2.5%. None of the 134 remaining phlebotomine sand fly pool samples showed any amplification product.

**Figure 3:**
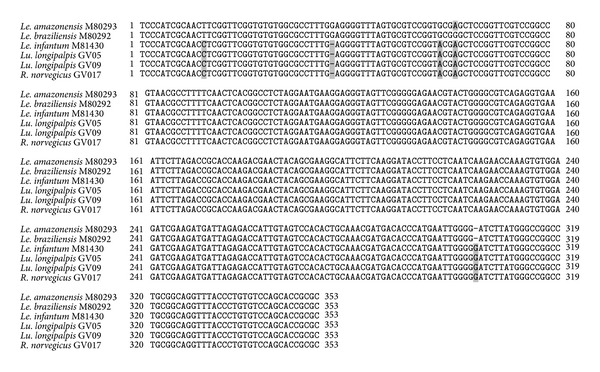
Nucleotide (nt) alignment of the 353 bp fragment of *Leishmania* spp. DNA from *Lu. longipalpis* (GV05 and GV09) and *R. norvegicus* (GV017) with *Le. (Viannia) braziliensis* (M80292.1), *Le. (V.) amazonensis* (M80293.1), and *Le. (Le.) infantum *(M81430.1) (syn *Le. chagasi*). Identical nucleotides are represented by dots and nucleotide deletion by a hyphen.

DNA sequencing of the 353 pb fragment followed by multiple alignment with database sequences for *Leishmania* species indicated *Le. infantum*, the etiological agent of VL, as the infecting agent in both pool samples of *Lu. longipalpis* (Figure 3).

Thirty-two small mammals, belonging to six distinct species, were captured during our study (Table 2). The most abundant species was *Rattus norvegicus* with almost 50% of the captured specimens. The 227 bp fragment that is characteristic of the IRBP gene was amplified in one hundred and nineteen blood and tissue samples from the small mammals captured (data not shown), indicating that DNA extractions were successful in every case. After LnPCR amplification, the presence of the characteristic 353 pb DNA fragment of *Leishmania* was verified in one among the 119 samples analysed. That single sample corresponded to blood DNA from a *R. norvegicus* specimen captured in January 2008 at the site identified in Figure 2. DNA sequencing and multiple alignments with data base sequences for *Leishmania* spp. indicated *Le. infantum*, the etiological agent of the VL, as the infecting parasite species in the *R. norvegicus* specimen (Figure 3). *Leishmania* spp. amastigotes were not observed in any of the 39 tissue imprints examined.

## 4. Discussion

In the present study we evaluated the epidemiological profile in an urban area endemic for ACL in relation to the vector, the hosts, and the parasite. Our entomological survey pointed to a high proportion of two ACL vector species, *Lu. intermedia* and *Lu. whitmani*. *Lu. intermedia* reached about 50% of all female sand flies. This finding is in accordance with another entomological survey also performed in Governador Valadares [16]. Although we did not identify DNA from any *Leishmania* species involved in ACL transmission in *Lu. intermedia* and *Lu. whitmani*, both species may be probably involved in the transmission of ACL in Governador Valadares. The primary vector of VL in Brazil, *Lu. longipalpis*, was also found, and its presence has also been observed before in the same municipality [16]. DNA from *Le. infantum*, the etiological agent of the same disease, was detected in two pool samples of this species. The TMI of 2.5% for *Le. infantum* in *Lu. longipalpis* is consistent with data described by others [17, 18].

The infection of domestic and wild mammals by *Le. infantum* has been studied in order to identify other possible reservoirs for VL, besides dogs [19]. Rodent species such as *Rattus rattus* (roof rat) and *R. norvegicus* (Norway rat), both nonnative species to South America but well adapted to human environment, stood out as possibilities [20]. Those species display consistent characteristics expected for reservoirs. Besides living in colonies, they are asymptomatic for leishmaniasis and have a sufficient longevity to maintain the infectious agent for the required period [21]. *R. rattus* was found naturally infected by *Le. donovani* and *Le. infantum* in the Old World [22–25] and by *Le. mexicana*, *Le. braziliensis*, and *Le. donovani* complexes in the New World [26–28]. Those findings indicate that *R. rattus *is a potential reservoir for both ACL and VL.

Although limited in numbers, previous studies suggested a possible role for *R. norvegicus* in the transmission cycle of ACL. Besides the identification of *Le. braziliensis* DNA in some rodent specimens [29], experimental inoculation with *Le. major* led to the development of infection [30]. The number of reports, most of them restricted to the Old World, is still smaller regarding *R. norvegicus* infection by *Le. infantum* [25, 31]. A seroprevalence of 33.3% was reported for *R. norvegicus* in Italy [25]. Differently, a much lower prevalence was found in Greece and the authors considered it unlikely that the rodent species might play any role as *Leishmania* reservoir [32]. Nevertheless, two important points were raised regarding low prevalence data of *Leishmania* infection in rodents [30]. Firstly, insufficient sensitivity of the tests used could account for an underestimation of the prevalence rates of *Leishmania* infection. Even if that situation is surpassed today by the introduction of more modern and sensitive techniques, a second important point still remains. In general, the rodents captured are adults and the susceptibility to infection may be restricted to young animals. Considering their continuous presence in the human environment due to the proximity of the borrows and to the all-year-round reproduction, the infection of younger rodent specimens, even if transient, might increase their role as *Leishmania* reservoirs [30].

Although our initial focus was the study of ACL in an endemic area, we identified the causative agent of VL (*Le. infantum*) in *Lu. longipalpis* and in *R. norvegicus* in Governador Valadares. The infected pool samples of *Lu. longipalpis* were captured in two distinct urban districts, one more central and the other closer to the Ibituruna's Peak (Figure 1). The urban characteristics of both districts testify the adaptation of *Lu. longipalpis* to environments modified by humans and the urban transmission pattern of VL in Governador Valadares. In a previous study of the local fauna, 90% of the phlebotomine sand flies captured in Governador Valadares were *Lu. longipalpis* [33]. The presence of *Le. infantum* DNA in this species of phlebotomine sand flies indicate the potential of VL transmission in the region.

VL is endemic in four of the five geographical regions of Brazil and, in recent years, it gained an increased importance in public health due to geographical expansion and urbanization [34, 35]. Dogs are considered the main domestic reservoirs of VL [36]. Wild *Lycalopex vetulus* and *Cerdocyon thous* canids and *Didelphis albiventris* marsupials were found naturally infected by *Leishmania* in the New World [36–38] whereas *Canis aureus* jackals, *Canis lupus* wolves, and *Vulpes vulpes* foxes have been described as wild hosts of VL in the Old World [39]. The presence of *Le. infantum* DNA in *R. norvegicus* is reported here for the first time in the New World and it seems possible that these rodents might play a role as VL reservoir. The parasite was detected only in blood not in skin samples. However, it is known that phlebotomine sand flies feed through a pool feeding mechanism and that VL transmission may occur if the parasite is present in the skin or in the peripheral blood of the reservoir [40]. Anyway, future studies will be needed to clarify any role played by *R. norvegicus* in VL transmission, especially regarding susceptibility and development of *Le. infantum* infection at different life cycle stages of that rodent species. *R. norvegicus* specimens accounted for almost 50% of the small mammals captured. Thus, the fact that we did not find *Leishmania* DNA in other species might be due to the small number of specimens captured from each one.

The Doce River Valley was considered endemic for ACL and VL in the past. After successful application of control measures for VL, the region was considered under control around the 80's [9]. The number of human VL cases was strongly reduced and restricted to non-autochthonous ones. In the beginning of the 90's, however, the control program was interrupted and the epidemiological surveillance was not regularly performed. In 2007, Malaquias et al. [41] suggested a possible reemergency of human VL in Governador Valadares based on the high prevalence of canine VL in the urban (13.7%) and rural (12.4%) areas and the confirmation of the parasite in seropositive dogs. Very recently, higher prevalences of canine VL were confirmed there by Barata et al. [33] reaching up to 53.4% depending on the district. Fifty percent of the seropositive dogs examined were symptomatic.

From 2001 to 2006, sixteen cases of human VL were confirmed in Governador Valadares. Between 2007 and 2013, that number increased ten times with 164 cases notified up to now. These data demonstrate the urgency of resumption of control measures in the municipality. According to the epidemiological criteria adopted by the Brazilian Ministry of Health, the average number of human cases ≥ 4.4 in the last five years characterizes intense transmission of VL and this is presently the case of Governador Valadares.

## Figures and Tables

**Table 1:** Female phlebotomine sand flies captured in Governador Valadares, state of Minas Gerais (Brazil) with HP light traps. The captures were performed in 2009 and 2010. After identification, the specimens were pooled according to species and capture site for molecular analysis.

Species	Females captured		Number of pools per species
	Number	Percentage	
*Lutzomyia cortelezzii *	63	10.4	27
*Lu. intermedia *	307	50.5	46
*Lu. ischyracantha *	42	6.9	11
*Lu. longipalpis *	78	12.8	21
*Lu. quinquefer *	65	10.7	18
*Lu. termitophila *	3	0.5	2
*Lu. whitmani *	50	8.2	11

Total	608	100	136

**Table 2:** Small mammals captured in Governador Valadares, state of Minas Gerais (Brazil) using Tomahawk traps. The captures were performed bimonthly from January to December of 2009 and from January to March of 2010.

Species	Common name	Specimens captured	
		Number	%
*Calomys *sp.	Cotton rat	1	3.1
*Didelphis albiventris *	The white-eared opossum	2	6.2
*Didelphis aurita *	The big-eared opossum	7	21.9
*Mus musculus *	House mouse	3	9.4
*Rattus norvegicus *	Norway or brown rat	15	46.9
*Rattus rattus *	Black rat	4	12.5

Total		32	100
